# Deficiency of the RNA-binding protein ELAVL1/HuR leads to the failure of endogenous and exogenous neuroprotection of retinal ganglion cells

**DOI:** 10.3389/fncel.2023.1131356

**Published:** 2023-02-17

**Authors:** Anna Pacwa, Joanna Machowicz, Saeed Akhtar, Piotr Rodak, Xiaonan Liu, Marita Pietrucha-Dutczak, Joanna Lewin-Kowalik, Marialaura Amadio, Adrian Smedowski

**Affiliations:** ^1^Department of Physiology, Faculty of Medical Sciences in Katowice, Medical University of Silesia in Katowice, Katowice, Poland; ^2^GlaucoTech Co., Katowice, Poland; ^3^College of Applied Medical Sciences, Inaya Medical Colleges, Riyadh, Saudi Arabia; ^4^Department of Optometry, College of Applied Medical Sciences, King Saud University, Riyadh, Saudi Arabia; ^5^Institute of Biotechnology, HiLIFE, University of Helsinki, Helsinki, Finland; ^6^Department of Drug Sciences, Section of Pharmacology, The University of Pavia, Pavia, Italy

**Keywords:** glaucoma, retinal ganglion cells (RGC), RNA-binding protein, HuR (ELAVL1), neuroprotection, AAV (adeno-associated virus)

## Abstract

**Introduction:**

ELAVL1/HuR is a keystone regulator of gene expression at the posttranscriptional level, including stress response and homeostasis maintenance. The aim of this study was to evaluate the impact of *hur* silencing on the age-related degeneration of retinal ganglion cells (RGC), which potentially describes the efficiency of endogenous neuroprotection mechanisms, as well as to assess the exogenous neuroprotection capacity of *hur*-silenced RGC in the rat glaucoma model.

**Methods:**

The study consisted of *in vitro* and *in vivo* approaches. *In vitro*, we used rat B-35 cells to investigate, whether AAV-shRNA-HuR delivery affects survival and oxidative stress markers under temperature and excitotoxic insults. *In vivo* approach consisted of two different settings. In first one, 35 eight-week-old rats received intravitreal injection of AAV-shRNA-HuR or AAV-shRNA scramble control. Animals underwent electroretinography tests and were sacrificed 2, 4 or 6 months after injection. Retinas and optic nerves were collected and processed for immunostainings, electron microscopy and stereology. For the second approach, animals received similar gene constructs. To induce chronic glaucoma, 8 weeks after AAV injection, unilateral episcleral vein cauterization was performed. Animals from each group received intravitreal injection of metallothionein II. Animals underwent electroretinography tests and were sacrificed 8 weeks later. Retinas and optic nerves were collected and processed for immunostainings, electron microscopy and stereology.

**Results:**

Silencing of *hur* induced apoptosis and increased oxidative stress markers in B-35 cells. Additionally, shRNA treatment impaired the cellular stress response to temperature and excitotoxic insults. *In vivo*, RGC count was decreased by 39% in shRNA-HuR group 6 months after injection, when compared to shRNA scramble control group. In neuroprotection study, the average loss of RGCs was 35% in animals with glaucoma treated with metallothionein and shRNA-HuR and 11.4% in animals with glaucoma treated with metallothionein and the scramble control shRNA. An alteration in HuR cellular content resulted in diminished photopic negative responses in the electroretinogram.

**Conclusions:**

Based on our findings, we conclude that HuR is essential for the survival and efficient neuroprotection of RGC and that the induced alteration in HuR content accelerates both the age-related and glaucoma-induced decline in RGC number and function, further confirming HuR’s key role in maintaining cell homeostasis and its possible involvement in the pathogenesis of glaucoma.

## 1. Introduction

Optic neuropathy is a common term for a group of neurodegenerative diseases related to retinal ganglion cell (RGC) death that may result in progressive and irreversible vision loss (Nuschke et al., 2015; Fan et al., 2019; Nakazawa and Fukuchi, 2020; Vernazza et al., 2021). RGCs provide a direct link between the retinal neuronal web and primary visual centers within the brain, and their axons, which form the optic nerve, represent the only route of visual signal transduction. Retinal neurons are designed to function in an unfavorable environment of pronounced phototoxic stress related to an increased oxidative status, leading to a high risk of cellular damage (Solley and Sternberg, 1999; Youssef et al., 2011; Ouyang et al., 2020; Upadhyay et al., 2020). Central nervous system neurons, including RGCs, represent non-proliferating post-mitotic cells that are unable to undergo spontaneous regeneration following damage (Moore and Goldberg, 2010; Calkins et al., 2017; Chun and Cestari, 2017; Laha et al., 2017; Varadarajan and Huberman, 2018; Quan et al., 2022). During their lifetime, RGCs are constantly exposed to exogenous stress and to a physiological, progressive aging-related impairment of their defense mechanisms (Pietrucha-Dutczak et al., 2018; Sanz-Morello et al., 2021). However, the adequate function of endogenous neuroprotective systems in RGCs ensures constant homeostatic maintenance during cell aging, provides cells with the appropriate response to damaging microenvironmental factors and, although not commonly emphasized, enables the proper activity of applied neuroprotective therapies (Liu and Greene, 2001; Osborne, 2008; Chrysostomou et al., 2010; Almasieh et al., 2012; Himori et al., 2013; Osborne and del Olmo-Aguado, 2013; Frade and Ovejero-Benito, 2015; Gauthier and Liu, 2016; Pietrucha-Dutczak et al., 2018; Naguib et al., 2021). Multiple mechanisms by which neurons maintain their intracellular homeostasis have been identified. Based on accumulating evidence, RNA-binding proteins contribute to endogenous neuroprotection and their alterations are linked to neurodegeneration (Gray and Woulfe, 2013; Szymanski et al., 2013; Sanna et al., 2014, 2016, 2017; Skliris et al., 2015; de Conti et al., 2017; Wang et al., 2017; Smedowski et al., 2018; Wei et al., 2019; Ambrosio et al., 2021). Among the best-known RNA-binding proteins is the Hu/ELAV-like (ELAVL) family (Skliris et al., 2015; Bowles et al., 2021), whose ubiquitously expressed member HuR (ELAVL1) undoubtedly plays role in the homeostasis and survival mechanisms of neurons, including those at the retinal level, serving as the master regulator of intracellular homeostasis pathways (Pascale and Govoni, 2012).

Retinal ganglion cell degeneration is a common process occurring at a slow rate over time that is physiologically related to aging (Frade and Ovejero-Benito, 2015). Additional external stimuli, such as ischemia, increased intraocular pressure (IOP) or inflammation, may accelerate this age-related cell death and, by impairing RGC function, lead to blindness. Neuroprotective mechanisms aim to limit the negative chain reactions induced by insult, prolonging the survival of RGCs (Doozandeh and Yazdani, 2016; Gauthier and Liu, 2016). Adequate neuroprotection involves multiple proteins and factors belonging to major families of stress response genes, i.e., *p53*, *sod*, and *hsp70*, and inflammation-controlling genes, i.e., *tnf*α and growth factors such as *bdnf*, *vegf*, and many others (Pietrucha-Dutczak et al., 2018). Interestingly, the ELAVL1 protein has been recognized as a factor regulating the expression of most stress response-related proteins, placing it in the position of a pivotal element in the chain of neuroprotective pathways (Pietrucha-Dutczak et al., 2018; Smedowski et al., 2018).

A deficiency of HuR protein in hippocampal neurons accelerates their death and, on the other hand, makes applied neuroprotective treatments ineffective (Skliris et al., 2015). In our previous studies, we described that HuR is the only representative of the ELAV family expressed in the retina; thus, its deficiency may be detrimental for RGC survival (Smedowski et al., 2018). Since we discovered that the HuR content is decreased in RGCs undergoing glaucomatous neurodegeneration, in the current study, we aimed to investigate whether silencing *hur* in healthy RGCs leads to accelerated glaucoma-like neurodegeneration. Additionally, we aimed to verify whether the lack of HuR renders the RGC neuroprotective approach ineffective. As a neuroprotective agent, we selected metallothionein II, which has been shown to exert recognized beneficial effects in many studies due to its antioxidant, antiapoptotic, and anti-inflammatory activities (Coyle et al., 2002; Penkowa, 2006; Suemori et al., 2006; Inoue et al., 2009; Pedersen et al., 2009a; Ling et al., 2016). We show here that AAV-based silencing of the *hur* gene induces retinal senescence in healthy rats and leads to pronounced RGC death in a glaucoma animal model, reflecting RGC function determined using electroretinography (ERG) and the RGC count measured in histology. Additionally, we report that intravitreally injected metallothionein II, which normally exerts beneficial effects on glaucomatous rat retinas, fails to protect RGCs when applied in *hur*-silenced animals.

## 2. Materials and methods

### 2.1. Animals and anesthesia

All experiments involving animals were approved by the Institutional Local Committee for Animal Research. Animals were treated in accordance with the European Communities Council Directive (86/609/EEC) and in a manner comparable to the guidelines published by the Institute for Laboratory Animal Research. Experiments were conducted in accordance with the ARVO Statement for the Use of Animals in Ophthalmic and Vision Research. In this study, we used 35 eight-week-old male Long Evans rats weighing approximately 200 g. During the experiments, animals were maintained on a 12-h dark-light cycle at a stable temperature and optimal humidity with free access to water and a standard pelleted diet. Each surgical procedure was performed under general anesthesia with an intraperitoneal injection of a mixture of ketamine (50 mg/kg; VetaKetam, Vetagro, Poland) and xylazine (5 mg/kg; Xylapan, Vetoquinol Biowet, Poland) and topical anesthesia with 0.5% proxymetacaine eye drops (Alcaine, Alcon, Fort Worth, TX, USA).

### 2.2. Design and validation of the shRNA construct

The shRNA constructs to silence the rat *hur* gene were purchased from Vigene Biosciences (Rockville, MD, USA). We tested the following four different plasmid sequences to identify the most efficient silencing construct: sh1-*GGAGGAACTACGGAGTCTGTT*, sh2-*GCCCAAGCTCAGAGG TTATCA*, sh3-*GCAGAAGAGGCAATTACCAGT*, and sh4-*GGTGCAGTTACCAATGTGAAA*. Rat neuroblastoma cells (B-35, ATCC, Manassas, VA, USA) were cultured in 6-well plates in Advanced DMEM (Gibco, Amarillo, TX, USA) supplemented with 10% fetal bovine serum (FBS, PanBiotech, Germany) and a 1% penicillin–streptomycin (P/S) solution (Gibco) at 37°C with 5% CO_2_. The volume of medium per well was 500 μL. After the cells reached 60% confluence, the transfection reagent was prepared by mixing 0.5 μg (3 μL) of each shRNA-GFP plasmid, 2 μL of Lipofectamine 2000 (Thermo, Waltham, MA, USA) and 100 μL of serum-antibiotic-free Advanced DMEM (Gibco). The transfection medium was placed in each well by combining 400 μL of antibiotic-free medium supplemented with 5% FBS and 100 μL of transfection reagent. After 24 h, the transfection efficiency was evaluated under a fluorescence microscope to determine the level of the intracellular GFP signal, and the transfection medium was replaced with fresh, antibiotic-free medium supplemented with 10% FBS. After an additional 24 h, the cells were collected and homogenized in a freshly prepared mixture of RIPA lysis buffer (Merck Millipore, Burlington, MA, USA) and Roche Complete Protease Inhibitor Cocktail (Roche, Switzerland). Lysates were separated by centrifugation at 4°C for 20 min and 12,000 rpm. The total protein concentration was measured with Bradford Reagent (Bio-Rad, Hercules, CA, USA) using the Quick Start Bovine Serum Albumin Standard Set (Bio-Rad) to obtain a standard curve. Samples were diluted to achieve a protein concentration of 2 mg/mL and denatured by adding Laemmli Sample Buffer (Bio-Rad) and boiling at 95°C for 5 min. Fifteen micrograms of protein concentrate were separated on 12% SDS–PAGE gels at 120 V and transferred onto PVDF membranes (Pall Life Sciences, New York, NY, USA) at 250 mA for 90 min. After transfer, the PVDF membranes were blocked with 3% BSA/0.1% Tween-TBS buffer for 1.5 h and incubated overnight at 4°C with the mouse ELAVL1/HuR antibody (dilution 1:1,000; Santa Cruz, Dallas, TX, USA). As a secondary antibody, we used HRP-conjugated goat anti-mouse (dilution 1:10,000, Abcam, UK). An HRP-conjugated alpha tubulin antibody (dilution 1:2,000, Rockland, Hercules, CA, USA) was used as a loading control. Signals on the membranes were detected using chemiluminescence (ChemiDoc MP, Bio-Rad). Protein bands were quantified using ImageJ software with the Band/Peak Quantification Tool.^1^

### 2.3. Design of the *in vitro* study

*In vitro* experiments aimed to investigate whether silencing the *hur* gene in B-35 rat neuroblastoma cells affects their survival under cytotoxic conditions induced by different insult conditions. Since the literature knowledge about *hur* silencing and neurons survival is limited, this part allowed us to perform screening safety evaluation of applied virus constructs before using them for *in vivo* purpose.

#### 2.3.1. B35 neuroblastoma cell culture and AAV-shRNA transfection

Experiments were performed using rat neuroblastoma cells (B-35, ATCC). Cells were cultured to 50% confluence in Advanced DMEM (Gibco) supplemented with 10% FBS (PanBiotech) and a 1% P/S solution (Gibco) at 37°C with 5% CO_2_. The medium was exchanged every 3rd day. After the cells reached 50% confluence, we performed AAV2-shRNA transfection (with a previously selected construct of shRNA-HuR or shRNA scrambled control). The transfection medium was prepared by mixing the AAV stock (calculated as 10^5^ AAV particles per single cell) with Advanced DMEM supplemented with 2% FBS and 1% P/S. Cells were incubated with the transfection medium for 36 h at 37°C with 5% CO_2_. Afterward, the transfection medium was replaced with fresh, fully supplemented culture medium (Advanced DMEM + 10% FBS + 1% P/S), and the cells were cultured for another 24 h. The transfection efficiency was evaluated by measuring the HuR protein content in fractionated Western blots.

#### 2.3.2. NMDA treatment and temperature insults

B-35 cells were grown in 6-well plates at a seeding density of 5 × 10^4^ cells per well. The cells were transfected as described above and assigned to six different study groups: treatment/insult (T/I) group, insult/treatment (I/T) group, preconditioning/insult (P/I) group, preconditioning/no insult (P) group, insult/no treatment (I) group, and control (C) group. The temperature insult (I) was optimized as an incubation at 48°C for 15 min with a subsequent 2-h incubation at 37°C. Preconditioning (P) was performed by applying 3 rounds of 15 min of incubation at 45°C every 2 h. Metallothionein II (AH Diagnostics, Helsinki, Finland, 1 μg/mL) was added to the culture medium for 2 h (T). The cells were then collected, homogenized, and fractionated to obtain cytoplasmic and nuclear fractions for subsequent Western blot experiments.

NMDA (Sigma, St. Louis, MO, USA) (10 or 20 mM) was added to the culture medium and incubated with the cells for 24 h. The experiment was conducted using 8-well cell culture chambers (Corning, Corning, NY, USA). We detected reactive oxygen species and apoptosis in cells after NMDA treatment by performing MitoSOX (Thermo) staining, the CaspACE assay (Promega, Madison, WI, USA) and immunostainings.

#### 2.3.3. AlamarBlue assay

We used the alamarBlue test (Thermo) to determine cell viability, which evaluates the rate of resazurin reduction according to the generation of fluorescence signals in proliferating cells, in order to find the cut-off dose of NMDA for further experiments. We prepared 5 different concentrations of NMDA by diluting the 63 mM stock solution in the supplemented medium. Twenty-four hours after virus transfection, the cells were exposed to NMDA (15, 20, 25, 30, or 35 mM) and incubated for an additional 24 h at 37°C with 5% CO_2_. Following this incubation, the culture medium was replaced with a mixture of 10% alamarBlue HS Cell viability reagent (Thermo) diluted in Advanced DMEM (Gibco), and the cells were incubated for 3 h at 37°C with 5% CO_2_. The cells were then transferred to a Spark microplate reader (Tecan, Switzerland), and the fluorescence signal was quantified at excitation and emission wavelengths of 570 and 630 nm, respectively.

#### 2.3.4. Apoptosis assay, oxidative stress markers, and immunostaining of cultured cells

Cells from the shRNA-HuR and shRNA-control groups were treated with either 10 or 20 mM NMDA and incubated at 37°C with 5% CO_2_ for 24 h to compare the molecular changes induced by the insults. After a 24-h exposure to NMDA, we performed a CaspACE apoptosis assay (CaspACE™ FITC-VAD-FMK *in situ* Marker, Promega). Cells were incubated with the marker at a final concentration of 10 μM for 20 min at 37°C with 5% CO_2_ in the dark. Centrifugation and washing steps were performed according to the manufacturer’s protocol. Suspended cells were seeded on a microscope slide, dried, and fixed with 4% ice-cold paraformaldehyde (PFA) for 30 min. The mean ratio of CaspACE-positive/DAPI cells was calculated from 5 visual fields using 200× magnification of fluorescent microscope.

For immunofluorescence staining, cells were rinsed with sterile 0.1 M PBS and fixed with 4% PFA for 1 h at 4°C. The cells were then washed 4 times with TBS and incubated with a blocking solution containing 10% NGS in TBST buffer (TBS + 0.1% Triton X) for 30 min. We used a rabbit primary antibody (dilution 1:300; Abcam) against 4-HNE, an oxidative stress marker for lipid peroxidation and Alexa Fluor 594-conjugated secondary antibody (1:500, Life Technologies, Carlsbad, CA, USA). The images quantification was performed using signal intensity analysis with imageJ software, averaged from 5 different photographs captured under 200× magnification of fluorescent microscope.

A MitoSOX (Thermo) assay was performed to detect reactive oxygen species in cells. Cells were incubated with 2 mM MitoSOX Green Reagent for 30 min at 37°C with 5% CO_2_, washed 3 times with sterile PBS and fixed with 4% PFA for 15 min at 4°C. The images quantification was performed as for immunostainings described above.

For the temperature insults, we did not perform immunostaining due to pronounced cell death in the shRNA-HuR groups that was already clearly visible under a light microscope.

For fluorescent microscopy, we used AxioScope.A1 (Zeiss, Germany) equipped with monochromatic AxioCam ICm1 Rev camera, 470/540–580 nm LED source and following filters, 38 HE eGFP, 43 HE Cy 3, 49 DAPI. The working optics included PL 10×/23 Br. foc. ocular lens and A-Plan 10×/0.25, A-Plan 20×/0.45, N-Achroplan 40×/0.65, N-Achroplan 100×/1.25 objectives. Images were processed with ZEN 2 Blue Edition, version 2.0.0.0 software. Images were captured using “auto exposure” mode and “auto best-fit” display. High resolution TIFF images were saved after scaling with software-inbuilt algorithm.

#### 2.3.5. Western blotting

Protein extracts from either total homogenates or subcellular fractions were used for Western blot assays with standard procedures. Nuclear and cytoplasmic fractions were separated using a Nuclear Extract Kit (Active Motif, Carlsbad, CA, USA) according to the manufacturer’s instructions. Fifteen micrograms of protein concentrate were separated on 12% SDS–PAGE gels at 120 V and transferred onto PVDF membranes (Pall Life Sciences) at 250 mA for 90 min. After transfer, the PVDF membranes were blocked with 3% BSA/0.1% Tween-TBS buffer for 1.5 h and incubated overnight at 4°C with the following primary antibodies: mouse ELAVL1/HuR (dilution 1:1,000; Santa Cruz) and mouse HSP70 (dilution 1:2,000; Santa Cruz). As a secondary antibody, we used an HRP-conjugated goat anti-mouse antibody (dilution 1:10,000, Abcam). An HRP-conjugated alpha tubulin antibody (dilution 1:2,000, Rockland) was used as a loading control. The membrane signals were detected using chemiluminescence (ChemiDoc MP, Bio-Rad). Protein bands were quantified using ImageJ software with the Band/Peak Quantification Tool (see text footnote 1).

### 2.4. Design of the *in vivo* study

#### 2.4.1. Age-related degeneration of RGCs—Endogenous neuroprotection

The main goal of this experiment was to determine whether AAV-based *hur* silencing accelerates the physiological age-related decline of RGCs. For this purpose, 18 animals were divided into 2 groups (*n* = 9 + 9) that received and intravitreal injection of either AAV-shRNA-HuR or AAV-shRNA-control in right eye; animals were then sacrificed at three different time points: 2, 4, and 6 months after the intravitreal AAV injection. Fellow eye of each animal was untreated and represented the healthy control. During the follow-up period, functional measurements ERG were performed. After the follow-up, the animals were sacrificed, and both healthy and treated retinas from each animal were collected and processed as whole mounts for immunostaining. RGCs were manually counted and compared between groups.

#### 2.4.2. Glaucoma model—Exogenous neuroprotection with metallothionein II treatment

This experimental approach was followed to investigate whether *hur* gene silencing leads to the impairment of metallothionein II (MT)-mediated neuroprotective properties in RGCs in a glaucoma model. For this purpose, 16 animals were divided into two groups: experimental (shRNA-HuR, *n* = 8) and control (shRNA-scramble control, *n* = 8) groups. All animals received an AAV injection into the right eye. Eight weeks after the AAV injection, we performed episcleral vein cauterization in the limbal area of the right eye to induce glaucoma. A decrease in the aqueous humor outflow to episcleral veins resulted in an elevated IOP. Simultaneously, following episcleral vein cauterization, rats from each group received an intravitreal injection of 1 μg/mL MT (3 μL per eye) into the right eye. The left eye was untreated. During the follow-up period, ERG measurements were performed. Animals were sacrificed after a follow-up time of 8 weeks, and the retina and optic nerves were processed for quantitative analyses of RGCs and axons, respectively.

#### 2.4.3. Intravitreal injections of AAV and MT

For the gene delivery we used AAV2 serotype that has a high tropism to RGC, long-term expression and low immunogenic profile. The high tropism of AAV2 could be due to the high expression of heparin sulfate proteoglycan by RGC, which mediates attachment of the AAV2 virus. After general and topical anesthesia was administered, the right eye of each animal was rinsed with a 10% povidone-iodine solution (Betadine, EGIS, Hungary). Animals from the experimental group received a 3 μL intravitreal injection of 10^8^ AAV-shRNA-HuR. The control group received 3 μL injections containing 10^8^ AAV-shRNA-scramble control. The injection was performed using a 5 μL Hamilton syringe with a 6 mm-long 34 G needle. The microneedle was gently introduced into the vitreous cavity, avoiding contact with the lens. After injection, the eye was topically treated with 2% chloramphenicol ointment (Detreomycin 2%, Chema-Elektromet, Poland) and secured with a clean dressing. Animals were housed singly in new cages for the next 24 h to avoid injury or infection after the procedure. The intravitreal injection of MT was performed in a similar manner as described above.

#### 2.4.4. Episcleral vein cauterization

For episcleral vein cauterization, animals were subjected to general and topical anesthesia. The curved pincet was gently inserted between the upper and lower eyelids, and the eyeball slightly protruded from the orbit. Four episcleral veins were identified and cauterized using thermal cautery (Faromed, Germany). Additional cauterization was performed on limbal vessels between episcleral veins after 4 weeks to produce a longer-lasting IOP elevation. After the procedure, the eye was topically treated with 2% chloramphenicol ointment (Detreomycin 2%, Chema-Elektromet) and secured with a clean dressing.

#### 2.4.5. Intraocular pressure measurements

The IOP was measured in both eyes of conscious animals before glaucoma induction, 1 day after the procedure and then once a week until the end of the experiment using a laboratory tonometer (TonoLab, Icare, Finland).

#### 2.4.6. ERG

During the *in vivo* study, electrophysiology tests were performed to evaluate the function of the retina. ERG was recorded using a Celeris system (Diagnosys LLC, UK). Animals underwent 12 h of dark adaptation before ERG tests. After dark adaptation, animals were anesthetized, pupils were dilated with 1% tropicamide eye drops (Polfa, Poland), and eyes were secured with moisturizing eye drops containing hyaluronic acid. Animals were placed on a heating pad throughout the recording session. We used a combined dark-light adaptation protocol that allowed us to evaluate the general condition of all retinal layers. The measurement conditions were 0.01 cds/m^2^, 0.1 cds/m^2^, 1.0 cds/m^2^, 3.0 flash, 10 flash, and 10 Hz flicker. We focused on a detailed analysis of negative photopic responses (PhNR) as a measure of RGC function. Measurements were performed at 3 time points of the endogenous neuroprotection experiment, 0, 2, and 6 months after AAV injection, and at 4 time points of the exogenous neuroprotection experiment: at the start point of the experiment, 8 weeks after virus injection (before glaucoma induction), 1 month after glaucoma induction and at the end point—2 months after glaucoma induction. For the PhNR analysis, we used a 10.0 flash to ensure a maximum photopic response.

#### 2.4.7. Animals’ euthanasia and tissue collection

Animals were euthanized by administering an overdose of anesthetic, and they were sequentially perfused through the left ventricle of the heart with 500 mL of 0.1 M PBS and 500 mL of 4% PFA for immunostaining or with 500 mL 0.1 M PBS followed by 500 mL 4% PFA/0.25% glutaraldehyde (GA) for electron microscopy. After isolation, the eyes were post-fixed with 4% PFA for immunostaining. Optic nerves were dissected and placed in 4% PFA/0.25% GA. After 6 h of fixation, retinas were removed from the eyeballs and subsequently fixed with 4% PFA overnight at 4°C.

#### 2.4.8. Immunostaining of rat retinas and optic nerves

Whole mounted retinas were placed into 48-well plates and washed 4 times in TBS. Tissues were incubated in a blocking solution containing 10% NGS in TBST buffer (TBS + 0.1% Triton X) for 30 min to eliminate non-specific binding. Subsequently, retinas were incubated with the primary antibody at + 4°C overnight and with secondary antibody for 3 h at RT. As a primary antibody, we used rabbit anti-RBPMS (dilution 1:500, Abcam) to visualize RGCs and mouse anti-HuR (1:300, Santa Cruz) to evaluate HuR expression after shRNA silencing. We used a rabbit anti-Iba1 antibody (1:500, Abcam) to evaluate inflammation within the retina. We used Alexa Fluor 488/594-conjugated secondary antibodies (1:500, Life Technologies). Nuclei were counterstained with 4′,6-diamidino-2-phenylindole (DAPI, Sigma–Aldrich). Samples were examined under a Zeiss Axioscope A1 fluorescence microscope (Zeiss), similarly as described in section “2.3.4. Apoptosis assay, oxidative stress markers, and immunostaining of cultured cells.”

Longitudinal paraffin sections of optic nerves were deparaffinized in xylene and hydrated in decreasing concentrations of ethanol and TBS. Tissue sections were then boiled in 0.01 M citric acid buffer, blocked with 10% NGS in TBST buffer and processed for immunostaining analogously to whole mounted retinas. For optic nerve sections, we used a primary mouse anti-Tuij-1 antibody (dilution 1:300, Santa Cruz) and rabbit anti-Iba1 antibody (1:500, Abcam). Nuclei were counterstained with 4′,6-diamidino-2-phenylindole (DAPI, Sigma–Aldrich).

#### 2.4.9. RGC and microglial cell counting

Retinal ganglion cell survival was evaluated by manually counting RBPMS-positive cells in the ganglion cell layer and microglia were evaluated for Iba-1 marker, using ImageJ software with the Multi-Point Tool. For the cell count, we used the corresponding half of each retina containing the superior and inferior quadrants. For a proper representation, we captured 12 corresponding fields from the central part of the retina (within 2 mm from the optic nerve output) and 12 from the peripheral part (greater than 2 mm distance from the optic nerve output) at 200× magnification. The mean number of cells in each group was calculated and presented as cell density per mm^2^.

#### 2.4.10. Electron microscopy

Eyes were processed for EM to evaluate the density of axons within optic nerves and to study the ultrastructure of optic nerves during the degeneration process. Optic nerves were fixed with 0.25% GA in 0.1 M PBS immediately after the death of the animal. The tissue was washed with 0.1 M PBS (3 × 15 min), post-fixed in osmium tetroxide for 1 h, washed with water (3 × 15 min) and dehydrated through a gradient of ethanol solutions (50–100%) for 15 min each followed by 100% acetone (2 × 30 min). Optic nerve stumps were infiltrated with a mixture of acetone and Spurr resin (1:1 and 1:2) for 2 h each. The tissue was subsequently infiltrated with 100% Spurr resin (3 × 8 h) and embedded in Spurr resin for 8 h at 70°C. Semithin (1 μm) and ultrathin (0.1 μm) sections were cut from blocks using an RMC ultracut microtome. All sections were cut as cross-sections within the myelinated part of the optic nerves. Semithin (1 μm) sections were collected on glass slides and stained with toluidine blue. The ultrathin sections were collected on 200 mesh copper grids. The sections were stained with 2% uranyl acetate (10 min) and lead citrate (10 min) and observed using a Jeol 1400 transmission electron microscope (Jeol Ltd., Akishima, Japan) using following parameters, HV 2100 kV, HFW 18.5 μm, Mag 3000×. Digital images were acquired using the iTEM program. The images were captured with a bottom mounted Quemesa camera. Axons were counted manually using ImageJ software from 10 corresponding visual fields representing the center and each quadrant of the optic nerve. The density of axons was shown per μm^2^.

### 2.5. Statistical analysis

For statistical analyses, we used Prism 9.3.1 software (GraphPad Software, Inc., La Jolla, CA, USA). Descriptive statistics are reported as the means ± standard deviations (SD). For the pairwise comparisons, we used Welch’s *t* test, which considers unequal SDs. For multiple comparisons, we used the Kruskal–Wallis test, and we applied the *post hoc* Bonferroni’s correction to pairwise comparisons within multiple groups. Correlations were determined by calculating Pearson’s correlation coefficients. *P*-values < 0.05 were considered significant.

## 3. Results

### 3.1. Evaluation of the shRNA silencing potency using the total HuR protein content

**FIGURE 1:**
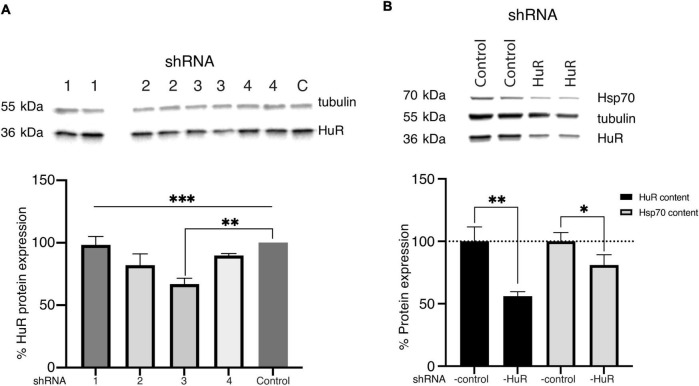
Validation of the shRNA constructs. **(A)** Western blot analysis (two biological replicates for each group) and representative images of the total HuR protein content in B-35 cell homogenates transfected with different shRNA-HuR constructs (Nuschke et al., 2015; Fan et al., 2019; Nakazawa and Fukuchi, 2020; Vernazza et al., 2021). Based on analysis, the construct sh3 showed the most silencing potency expressed as lowest HuR protein content. C—shRNA-control, ***p < 0.003. **p < 0.01. **(B)** Western blot analysis (two biological replicates for each group) of the silencing efficacy of the sh3RNA-HuR (sh3-GCAGAAGAGGCAATTACCAGT) construct; shRNA Control–cells after shRNA-scrambled control transfection, shRNA HuR–cells after shRNA-HuR transfection. The silencing potency was analyzed as the total HuR and Hsp70 protein contents in B35 cell homogenates following transfection. **p < 0.01; *p < 0.03.

In the homogenates of B-35 cells exposed to either the vehicle or four different shRNA-HuR constructs tested individually, there was a significant difference between tested constructs (*p* < 0.003, Kruskal-Wallis test) and the sequence sh3-*GCAGAAGAGGCAATTACCAGT* produced the most significant decrease in the total HuR protein level analyzed using Western blotting (Welch’s *t*-test, *p* < 0.01) (Figure 1A). After the most efficient construct had been identified, it underwent paired testing with an shRNA-scrambled control construct, to evaluate the impairment of HuR and Hsp70 expression, whose mRNA is a well-known target of HuR. In this analysis, the total HuR protein content was decreased by almost half after silencing (100 ± 11.6 vs. 56 ± 3.64%, Welch’s *t*-test, *p* < 0.01), and the total Hsp70 protein content was reduced by approximately 19% (100 ± 7.02 vs. 81.1 ± 8.2%, Welch’s *t*-test, *p* < 0.03) (Figure 1B). The reduction in the Hsp70 protein content provided further proof of efficient *hur* gene silencing in B-35 cells.

### 3.2. The *in vitro* study

#### 3.2.1. *hur* silencing alleviates the effects of cytoprotective treatments on B-35 cells exposed to high temperature and NMDA insults

**FIGURE 2:**
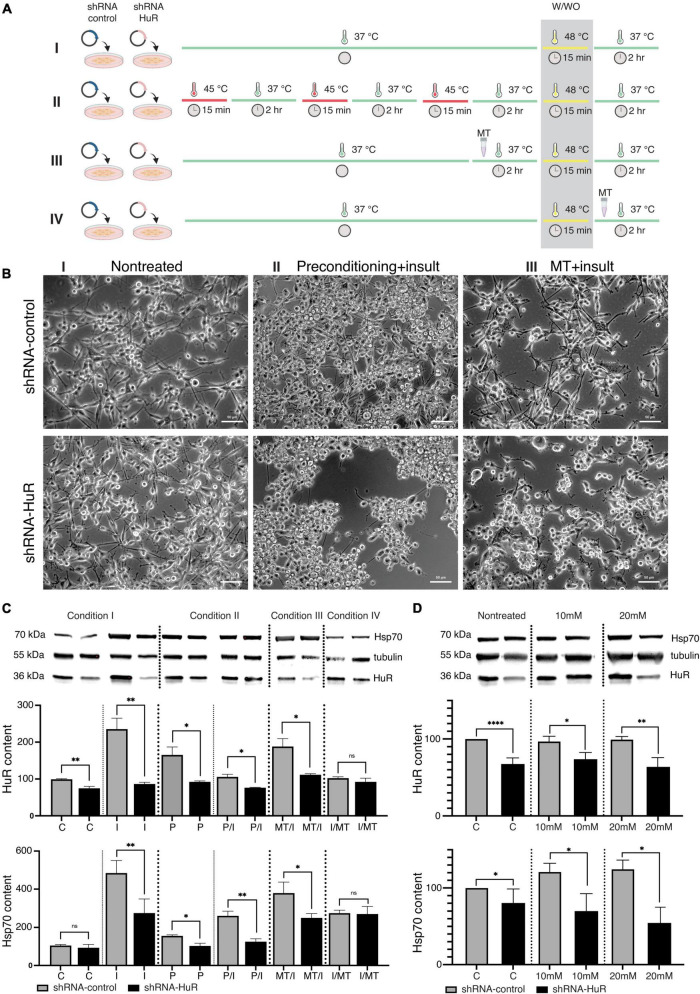
*In vitro* validation experiment using two cell death models, temperature insult and NMDA excitotoxicity in B35 cells. **(A)** Scheme of the *in vitro* temperature insult experiment performed in B-35 cells. Condition I—cells underwent transfection with either shRNA-HuR or shRNA-control: “C”—control group was cultured in standard medium and standard temperature condition; “I”—insult group was cultured in standard medium and was exposed to 48°C for 15 min. Condition II—cells underwent transfection with either shRNA-HuR or shRNA-control, then cells were exposed to temperature preconditioning by applying 3 rounds of 15 min of incubation at 45°C every 2 h, then one group was cultured in standard medium and standard temperature condition (“P” group) and the other group was exposed to 48°C for 15 min insult (“P/I” group). Condition III—cells underwent transfection with either shRNA-HuR or shRNA-control, then cells were treated with 1 μg/mL of MT for 2 h, following exposition to 48°C for 15 min insult (“T/I” group). Condition IV—cells underwent transfection with either shRNA-HuR or shRNA-control, then cells were exposed to 48°C for 15 min insult, following treatment with 1 μg/mL of MT for 2 h (“I/T” group). Condition IV did not exert any visible preventive effects on cells, and it was used solely as a reference group for WB analysis. **(B)** Light microscopy images of cultured B-35 cells exposed to the two preventive approaches, either temperature preconditioning or MT treatment, followed by high-temperature insult. Condition IV (not shown in photographs) did not exert any visible effects on cells, and it was used solely as a reference group for WB analysis. Scale bar = 50 μm. **(C)** Western blot analysis (two biological replicates for each group) and representative images of cytoplasmic HuR and Hsp70 protein levels; shRNA-control- and shRNA-HuR-treated B-35 cells were exposed to either preconditioning or MT before or after the insult (heat shock), as described above. **p < 0.01, *p < 0.03, and ns, not significant. **(D)** shRNA-control- and shRNA-HuR-treated B-35 cells were exposed to two different concentrations of NMDA, 10 or 20 mM for 24 h, in order to investigate the impact of excitotoxic insult on the cells with HuR deprivation; “C”—cells transfected with shRNA-control or shRNA-HuR with no insult applied; “10 mM,” “20 mM”—cells treated with either constructs + 10- or 20-mM NMDA. ****p < 0.001, **p < 0.01, and *p < 0.03.

B-35 cells were exposed to two preventive approaches before stress treatment: the first was represented by temperature preconditioning (P), aiming to induce an Hsp system response, while the second (T) was represented by metallothionein II (MT), an antioxidative stress protein that aims to alleviate the negative effects of the high-temperature insult (I) (Figure 2A).

In exploratory studies using a repeatable alamarBlue assay, we optimized the parameters of both the preconditioning temperature and the heat shock insult. The threshold temperature that did not affect B-35 cell survival but activated Hsp70 was 45°C for 15 min, while the condition inducing cell death was a 48°C incubation for 15 min. The selected working concentration of MT was 1 μg/mL applied for 2 h.

Silencing of the *hur* gene reduced the effects of both preventive approaches applied before the temperature insult, which was visible under a light microscope (Figure 2B). In WB analysis, significant differences in the cytoplasmic HuR and Hsp70 protein contents were observed (Figure 2C). The shRNA-HuR treatment resulted in decreased HuR levels (*p* < 0.03, Welch’s *t*-test) and a substantially impaired cellular response to insult. Preconditioning significantly increased the HuR content, which may be interpreted as the preparation of cells for further insult. As expected, this phenomenon was not observed in *hur*-silenced cells, and it was accompanied by pronounced cell death (Figure 2B). When MT treatment was applied for 2 h after the insult (15 min at 48°C) as a rescue approach, it did not affect the HuR content in any of the groups (*p* > 0.05, Welch’s *t*-test; last pair in Figure 2C), in contrast to MT treatment before insult, which led to an increase in the HuR content in the shRNA-scrambled control group and a significantly impaired HuR response in the shRNA-HuR group (*p* < 0.03, Welch’s *t*-test). In the experimental setting with the applied treatment (both MT and preconditioning), the *hur* silencing impaired the cellular response to the insult despite the neuroprotective strategy applied (both treatments resulted in significantly reduced HuR content, *p* < 0.03, Welch’s *t*-test). A similar trend was observed for Hsp70, although a larger difference was visible in groups with applied preconditioning (Figure 2C). In terms of NMDA-mediated excitotoxicity, *hur* gene silencing led to the inability of cells to generate a stress response, as evidenced by the low contents of both the HuR and Hsp70 proteins (Figure 2D).

#### 3.2.2. *hur* silencing accelerates B-35 cell death associated with oxidative stress induced by NMDA toxicity

**FIGURE 3:**
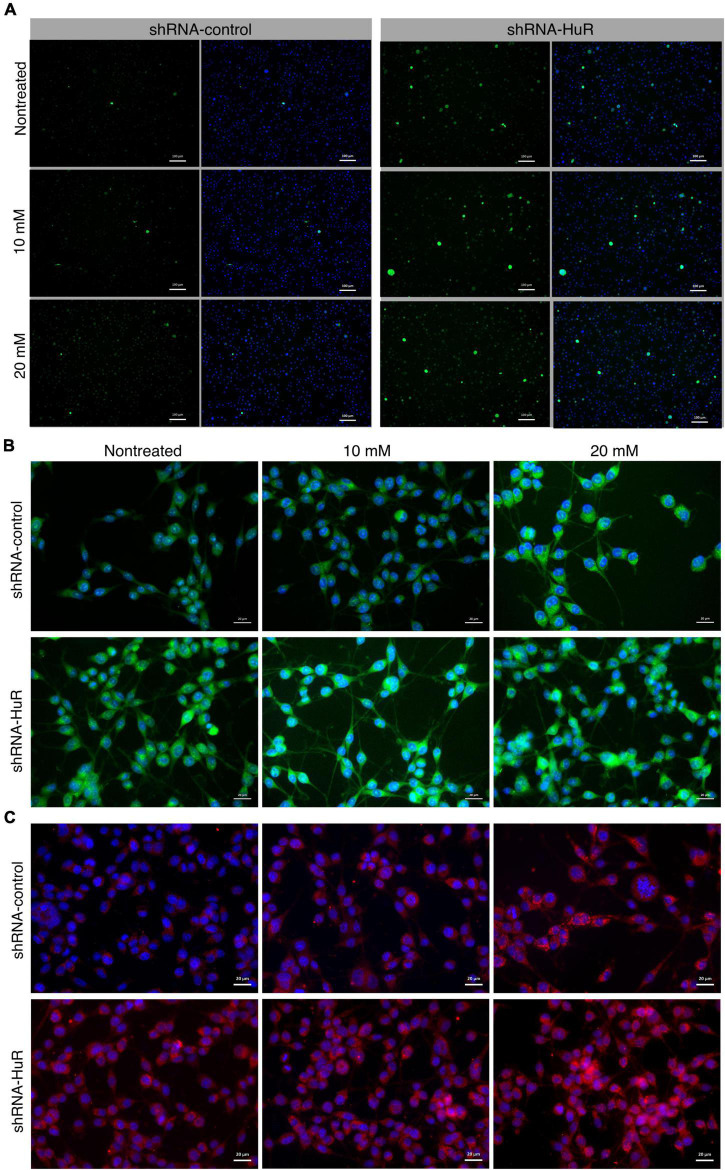
*In vitro* validation of oxidative stress and apoptosis using NMDA excitotoxicity model in B35 cells. **(A)** Fluorescence microscopy images of apoptotic cells detected using the CaspACE assay in shRNA-control- and shRNA-HuR-transfected B-35 cells exposed to NMDA (10 or 20 mM) for 24 h. Scale bar = 100 μm. CaspACE (green), DAPI (blue). **(B)** Fluorescence microscopy images of shRNA-control- and shRNA-HuR-treated B-35 cells exposed to NMDA (10 or 20 mM) for 24 h and then stained with MitoSOX Green marker of ROS (green); signals merged with DAPI staining (blue) are shown. Scale bar = 20 μm. **(C)** Fluorescence microscopy images of shRNA-control- and shRNA-HuR-treated B-35 cells exposed to NMDA (10 or 20 mM) for 24 h and stained for 4-hydroxynonenal (4-HNE, red); images of signals merged with DAPI staining (blue) are shown. Scale bar = 20 μm.

In both, the shRNA-control and shRNA-HuR groups, NMDA treatment increased the number of CaspACE-positive cells and the apoptosis index. The ratio of CaspACE-positive/DAPI cells was 0.5 ± 0.02, 1.1 ± 0.04, and 4.5 ± 0.9% per visual field under 200× magnification for shRNA-control, shRNA-control + 10 mM NMDA and shRNA-control + 20 mM NMDA, respectively. This apoptotic effect was much more evident in the shRNA-HuR group and the mean ratio of CaspACE-positive/DAPI cells was 2.5 ± 1.1, 9.6 ± 1.8, and 17.4 ± 4.3%, for shRNA-HuR, shRNA-HuR + 10 mM NMDA and shRNA-HuR + 20 mM NMDA, respectively (Figure 3A). The level of oxidative stress was evaluated using the MitoSOX assay and immunostaining for 4-hydroxynonenal (4-HNE), a cellular marker of lipid peroxidation. In these analyses, we observed evidence of increasing ROS and lipid peroxidation levels after treatment with increasing concentrations of NMDA; however, these changes were visibly more pronounced in shRNA-HuR-transfected cells than in controls (Figures 3B, C). For MitoSOX assay, the measured fluorescence intensities were 14.1 ± 2.4, 15.1 ± 2.6, and 16.3 ± 3.2 for shRNA-control, shRNA-control + 10 mM NMDA and shRNA-control + 20 mM NMDA, respectively and 15.3 ± 3.7, 16.2 ± 1.05, and 20.4 ± 5.3 for shRNA-HuR, shRNA-HuR + 10 mM NMDA and shRNA-HuR + 20 mM NMDA, respectively. In HNE staining, the mean measured fluorescence intensities were 16.8 ± 3.7, 16.9 ± 3.9, and 18.1 ± 4.3 for shRNA-control, shRNA-control + 10 mM NMDA and shRNA-control + 20 mM NMDA, respectively and 18.7 ± 4.1, 21.2 ± 5.2, and 28.6 ± 5.9 for shRNA-HuR, shRNA-HuR + 10 mM NMDA and shRNA-HuR + 20 mM NMDA, respectively.

### 3.3. The *in vivo* study

#### 3.3.1. *hur* silencing hyperaccelerates age-related RGC death

**FIGURE 4:**
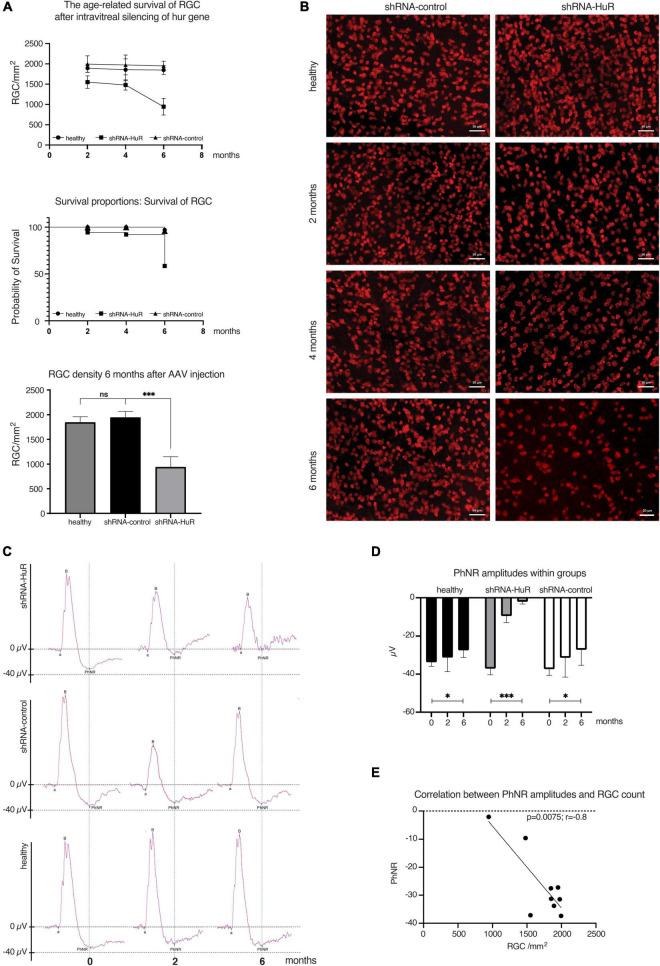
Age-related degeneration of RGCs—endogenous neuroprotection study. **(A)** Analysis of the RGC counts, survival curve and significance of RGC loss between groups of animals 2, 4, and 6 months after intravitreal injection of AAV constructs; *n* = 3 animals per group, 12 micrographs per retina. ns, not significant, ***p < 0.003. **(B)** Immunofluorescence staining of whole-mounted retinas for RBPMS (RGC marker) in animals sacrificed 2, 4, and 6 months after intravitreal injection of AAV constructs. Morphological differences are clearly visible, especially after 6 months, where in shRNA group, the cell loss seemed to be involving especially small RGC, so there was a higher content of larger-sized RGC observed, when compared with control group. Scale bar = 50 μm. **(C)** Averaged recordings of photopic ERGs with an analysis of the photopic negative response (PhNR) wave 2 and 6 months after AAV constructs injection. **(D)** Statistical analysis of changes in the PhNR amplitude within groups 2 and 6 months after AAV constructs injection; *n* = 3 animals per group. *p < 0.03 and ***p < 0.003. **(E)** The correlation analysis between RGC count (morphology) and PhNR responses (function) in animals utilized in experiment. r, correlation coefficient.

In rats exposed to either shRNA-HuR or shRNA-control that were evaluated 2, 4, or 6 months after the shRNA injection, the RGC count per mm^2^ was 1550 ± 155, 1480 ± 125, and 945 ± 205 cells for the shRNA-HuR group and 1995 ± 205, 1975 ± 245, and 1950 ± 115 cells for the shRNA-control group, respectively. In comparison, healthy eyes had average counts of 1895 ± 100, 1855 ± 270, and 1850 ± 110 RGCs per mm^2^ at the follow-up time points of 2, 4, and 6 months, respectively (Figure 4A). After 6 months, the loss of RGCs was equal to 2.3% for the shRNA-control group, 2.4% for healthy eyes (*p* > 0.05, Welch’s *t*-test with the *post hoc* Bonferroni’s correction) and 39% for the shRNA-HuR group (*p* < 0.003 when compared with both healthy and shRNA-control groups, Welch’s *t*-test with the *post hoc* Bonferroni’s correction). Morphological differences were clearly observed in the retinal whole mounts immunolabeled for RBPMS, especially after 6 months, where in shRNA group, the cell loss seemed to be involving especially small RGC, so there was a higher content of larger-sized RGC observed, when compared with control group (Figure 4B). The morphological decrease in the RGC count was associated with impaired function measured with photopic negative responses (PhNR) in ERG. After 6 months, the age-related loss of RGC function was 18.6% in healthy eyes (*p* < 0.03, Kruskal–Wallis test), 27.3% in shRNA-control-treated eyes (*p* < 0.03, Kruskal–Wallis test) and 94.3% in eyes injected with shRNA-HuR (*p* < 0.003, Kruskal–Wallis test) compared with initial recordings obtained at the beginning of the experiment (Figures 4C, D). We correlated the RGC count with PhNR amplitudes to evaluate whether the morphological changes accompany RGC function, and we found a highly significant association (*p* = 0.0075, *r* = −0.8, Pearson’s correlation coefficient, Figure 4E).

#### 3.3.2. *hur* silencing alleviates the neuroprotective properties of MT in a rat glaucoma model

**FIGURE 5:**
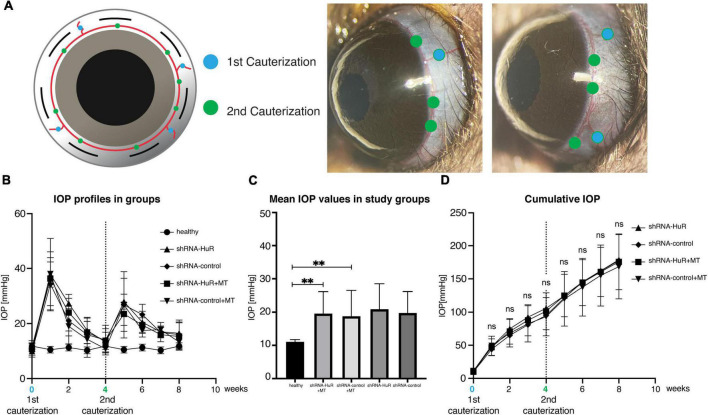
Glaucoma model. **(A)** The methodology of exogenous neuroprotection study—the thermocoagulation of episcleral and limbal vessels in rat eye (blue marker identifies episcleral veins, green marker—limbal vessels). Four episcleral veins were cauterized using thermal cautery (1st cauterization). Additional cauterization was performed on limbal vessels between episcleral veins after 4 weeks to produce a longer-lasting IOP elevation (2nd cauterization). **(B)** IOP profiles of the groups after 1st and 2nd cauterization. **(C)** The mean IOP induced in experimental groups (shRNA-HuR and shRNA-control) were significantly higher when compared with healthy eyes. **(D)** Cumulative IOP induced in glaucoma model. **p < 0.01, ns, not significant.

The rat glaucoma model was induced by thermocoagulation of episcleral and limbal vessels at weeks “0” and “4” to ensure a significant increase in IOP for an 8-week period (Figure 5A). The IOP values were similar for both experimental groups, shRNA-HuR + MT and shRNA-control + MT (Figure 5B), and on average, they were significantly higher than in healthy eyes (*p* < 0.001, Welch’s *t*-test, Figure 5C). The average cumulative IOP was comparable in both experimental groups (*p* > 0.05, Welch’s *t*-test) (Figure 5D). As reference groups, we used shRNA-HuR and shRNA-control groups that were not treated with MT. For these groups, thermocoagulation of episcleral veins resulted in IOP profile comparable with MT-treated animals (Figures 5B–D).

**FIGURE 6:**
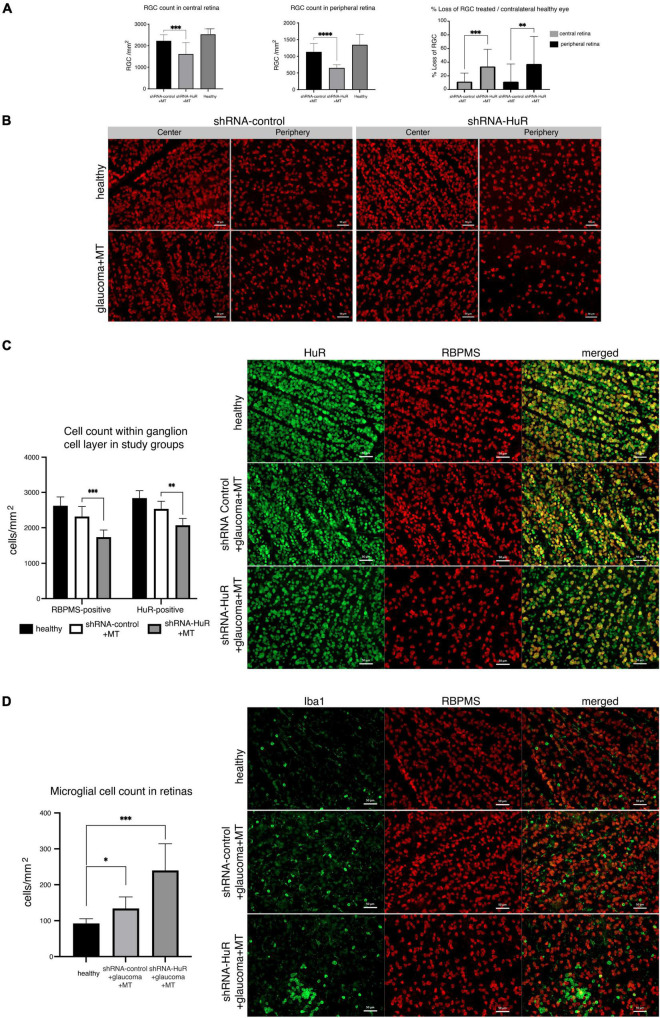
The exogenous neuroprotection study. **(A)** RGC counts in corresponding central and peripheral regions of whole-mounted retinas 8 weeks after glaucoma model induction and MT treatment, and % RGC loss in these retinas, *n* = 8 animals per group, 12 micrographs for each, central and peripheral retina. **p < 0.01, ***p < 0.003, and ****p < 0.001. **(B)** RBPMS immunofluorescence staining of the retinal center and periphery 8 weeks after glaucoma model induction and MT treatment. Scale bar = 50 μm. **(C)** Immunofluorescence staining for HuR (green) merged with RBPMS (red) and cell quantification in central region of whole-mounted retina. A visible difference in HuR expression is noticeable in the ganglion cell layer of retinal whole-mounts, although the loss of RBPMS-positive cells was greater than HuR-positive cells. **p < 0.01, ***p < 0.003. Scale bar = 50 μm. **(D)** Immunofluorescence staining for Iba-1 (green) merged with RBPMS (red) and cell quantification in central regions of whole mounted retinas. The infiltration of Iba-1-positive immune cells is particularly pronounced in the shRNA-HuR + MT group, whose microglial cells represented the morphological features of stimulated macrophages. *p < 0.03, ***p < 0.003. Scale bar = 50 μm.

In the glaucoma experiment, 8 weeks of increased IOP in rats induced a significant reduction in RGC counts (Figure 6A). In the retinal center, the RGC counts were 2537 ± 245, 2233 ± 280, and 1624 ± 525 per mm^2^ for the healthy, shRNA-control + MT, and shRNA-HuR + MT groups, respectively. The cell count observed after the shRNA-HuR injection was significantly lower than that in the shRNA-control group (*p* < 0.003, Welch’s *t*-test), although both groups received neuroprotective MT treatment and displayed comparable IOP levels. In the retinal periphery, the RGC counts were 1349 ± 310, 1135 ± 245, and 655 ± 91 per mm^2^ for the healthy, shRNA-control + MT control, and shRNA-HuR + MT groups, respectively. Notably, in this anatomical area, a more pronounced difference in RGC counts was observed between the shRNA-HuR + MT and shRNA-control + MT groups (*p* < 0.001, Welch’s *t*-test). Compared with healthy contralateral eyes, after 8 weeks of glaucoma and MT treatment, the RGC loss in the retinal center was 12% in the shRNA-control group compared with 36% in the shRNA-HuR group, while in the retinal periphery, it was 16% in the shRNA-control group compared with 38% in the shRNA-HuR group. The differences in RGC numbers were noticeable in the retinal whole-mounts immunolabeled for the RGC marker RBPMS (Figure 6B). A visible difference in HuR expression was also noted in the ganglion cell layer of retinal whole-mounts, although the loss of RBPMS-positive cells (33.7%) in shRNA-HuR + MT group was greater than loss of HuR-positive cells (27%) (Figure 6C). The glaucoma model, as well as the injection procedure itself, were associated with an increased presence of Iba-1-positive microglial cells in the retina. The density of Iba-1-positive cells were 92.5 ± 15, 134.3 ± 32, and 240 ± 74 cells per mm^2^ for healthy, shRNA-control + MT and shRNA-HuR + MT groups, respectively (Figure 6D). Infiltration of immune cells was the most significantly pronounced in shRNA-HuR + MT (*p* < 0.003, Welch’s *t*-test) and they presented the morphological features of stimulated macrophages (Figure 6D).

**FIGURE 7:**
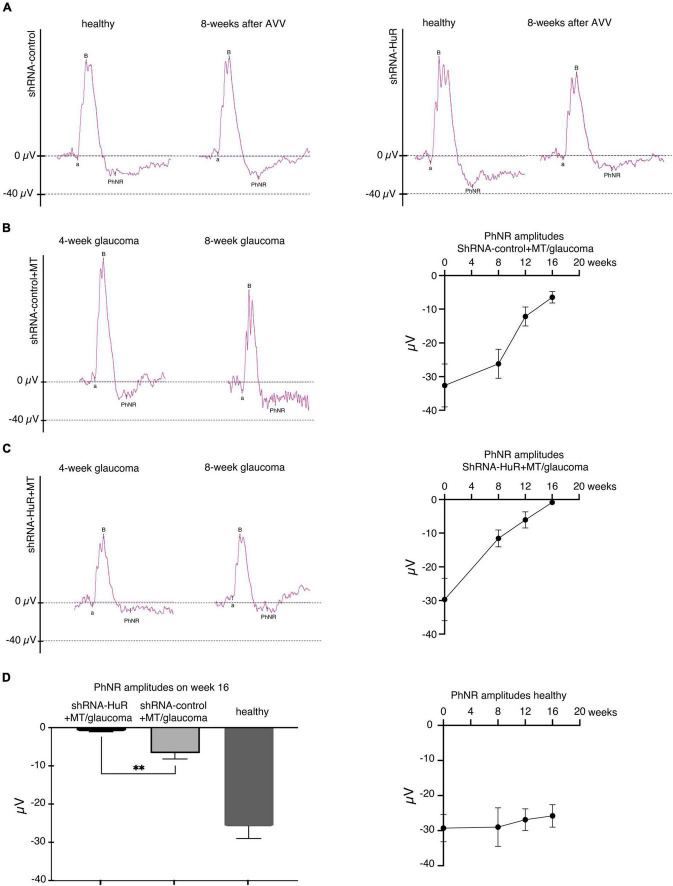
The exogenous neuroprotection study, functional analysis of RGCs based on the PhNR wave. **(A)** Silencing of the *hur* gene using shRNA-HuR leads to the acceleration of the loss of RGC function that is not observed after shRNA-control injection. **(B)** The effects of glaucoma and MT treatment on RGC function after the shRNA-control injection. **(C)** The effects of glaucoma and MT treatment on RGC function after the shRNA-HuR injection. Silencing of *hur* gene leads to almost complete loss of PhNR response despite of applied MT neuroprotective treatment. **(D)** Statistical analysis of PhNR wave amplitudes 16 weeks after AAV constructs injection, *n* = 8 animals per group, **p < 0.01.

In terms of functional measurements, the PhNR showed significant variations, especially at the end of the experiment (week 16 of follow-up, Figures 7A–C). The changes in PhNR wave amplitudes from week 0 to week 16 were −29.3 ± 3.9 to −25.8 ± 3.2; −32.6 ± 6.4 to −6.5 ± 1.7; and −29.7 ± 6.3 to −0.9 ± 0.1 μV for the healthy, shRNA-control + MT, and shRNA-HuR + MT groups, respectively. Based on the statistical analysis, a significant difference was observed between the shRNA-control + MT group and the shRNA-HuR + MT group at week 16 (*p* < 0.01, Welch’s *t*-test, Figure 7D).

**FIGURE 8:**
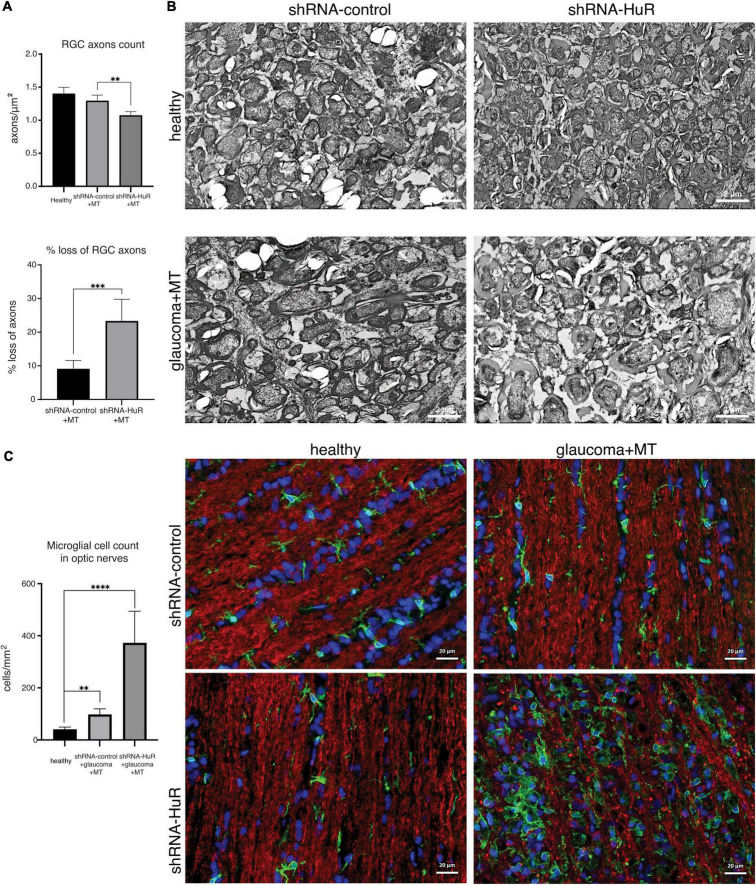
The exogenous neuroprotection study. Optic nerve histology analysis. **(A)** Optic nerve axon count and estimated loss between groups based on electron microscopy of optic nerve cross sections, *n* = 5 animals per group, 10 ultramicrographs per optic nerve, **p < 0.01, ***p < 0.003. **(B)** Representative electron micrographs of optic nerve cross sections, scale bar = 2 μm. **(C)** Immunofluorescence staining of optic nerve longitudinal sections for Iba-1 (green) merged with Tuij-1 (red) and DAPI (blue) and cell quantification. The pattern of Iba-1-positive macrophage infiltration was similar to that observed in the retinal whole-mounts. **p < 0.01, ****p < 0.001. Scale bar = 20 μm.

We performed an EM analysis and axon density per μm^2^ on optic nerve cross-sections to confirm the ultrastructural features of neurodegeneration (Figures 8A, B). In this analysis, a quantitative difference in axon density was observed between the shRNA-control + MT and shRNA-HuR + MT glaucoma groups (1.3 ± 0.08 axons per μm^2^ vs. 1.08 ± 0.05 axons per μm^2^; *p* < 0.01, Welch’s *t*-test). The average loss of axons in the shRNA-control + MT and shRNA-HuR + MT groups was estimated as 9.2 and 23.4%, respectively, compared to healthy contralateral eyes (*p* < 0.003, Welch’s *t*-test). The qualitative analysis of EM images revealed pronounced axon swelling and myelin sheet disintegration in the shRNA-HuR group, features that were not perceivable in shRNA-control animals (Figure 8B). In images of immunofluorescence staining of longitudinal sections of optic nerves, we observed a pattern of Iba-1-positive macrophage infiltration similar to that observed in the retinal whole-mounts (Figure 8C). The Iba-1-positive cell count was 41 ± 8, 98 ± 21, and 373 ± 121 cells per mm^2^, for healthy, shRNA-control + MT and shRNA-HuR + MT group, respectively. Similarly, as it was observed in the retina, the most intense immune cell infiltration was present in shRNA-HuR + MT (*p* < 0.001, Welch’s *t*-test, Figure 8C).

## 4. Discussion

Natural RGC degeneration appears as an aging-related process that physiologically progresses slowly, but it may be accelerated by additional factors, such as ischemia, an increased IOP or inflammation, leading to visual impairment and even blindness (Chrysostomou et al., 2010; Almasieh et al., 2012; You et al., 2013; Völgyi, 2020). In addition to the external detrimental stimuli modulating RGC function, the identification and characterization of endogenous neuroprotective mechanisms designed to maintaining homeostasis and ensure RGC defenses under stress are of key importance (Pietrucha-Dutczak et al., 2018). Indeed, the effective functioning of RGCs and, more broadly, the visual signal transduction pathway strictly depends on endogenous capacities to preserve their own health and to properly face and respond to unfavorable environmental stimuli (Pérez de Lara et al., 2014; della Santina and Ou, 2017; Fry et al., 2018). Similar to other post-mitotic cells that are unable to undergo spontaneous regeneration, the RGC response to cellular damage is the difference between preserved homeostasis and a drift toward progressive neurodegenerative phenotypes (Liu and Greene, 2001; Moore and Goldberg, 2010; Frade and Ovejero-Benito, 2015; Hu, 2016; Chun and Cestari, 2017; Chi et al., 2018; Quan et al., 2022). In this context, the present *in vivo* study indicates that the HuR/ELAVL1 protein is an indispensable factor for RGC homeostasis and the stress response (Smedowski et al., 2018). In healthy rats, *hur* silencing affected the RGC count in an age-dependent manner, with a consistent and accelerating decrease in cell viability mainly observed at 6 months after the intravitreal injection of shRNA-HuR; no significant RGC loss was detected in the untreated and shRNA-control groups. An early, substantial decrease in the PhNR amplitude, an index of RGC functionality, precedes RGC loss and is visible at 2 months after shRNA-HuR administration; the PhNR amplitude is completely lost at 6 months, an effect that strongly correlates with RGC death. These findings highlight two main aspects. First, at month 2, although the RGC count in the shRNA-HuR group was slightly lower than that in both the healthy and shRNA-control groups, RGC function was already impaired by approximately 70%. Second, when the RGC count was decreased by half, RGC function was considerably suppressed. Overall, these data suggest that a normal HuR protein content within RGCs contributes to both survival and maintenance of proper neurotransmission and, more generally, to preservation of retinal function in the context of the physiological age-dependent decline in functionality. In other words, and according to previous observation of HuR as a pro-juvenile factor in senescence, *hur* silencing confers a senescent phenotype to the rat retina, as displayed through early, progressive RGC loss and functional impairment (Wang et al., 2001; Abdelmohsen et al., 2008; Masuda et al., 2012). Since HuR is a keystone regulating the expression of a large number of genes at the post-transcriptional level, ranging from splicing to translation, the effects of *hur* silencing may be a consequence of altered RNA homeostasis. In particular, among HuR-controlled genes, many encode stress response proteins and factors involved in neuronal maintenance, such as Hsp70, HO-1, SOD1, BDNF, and VEGF (Amadio et al., 2008; Milani et al., 2013; Zhang et al., 2021). Moreover, since HuR interacts with not only mRNAs but also non-coding RNAs and other RNA-binding proteins, each is involved in the regulation of a broad spectrum of cellular processes (Kim et al., 2017), and any perturbation in HuR protein levels reverberates to the delicate balance of post-transcriptional controllers, a top cascade of intracellular events with potentially unlimited outcomes.

We previously showed that in rats subjected to intracameral injection of polystyrene microbeads, glaucomatous degeneration of the retina is accompanied by an altered HuR content 8 weeks later (Smedowski et al., 2018). The current study, which was performed in another rat glaucoma model, where an increased IOP is induced by double thermocoagulation of episcleral and limbal vessels on weeks “0” and “4,” confirms that RGC death is coupled with a decrease in HuR levels in the rat retina on week 8. The *hur* silencing-mediated effects on both shRNA-control and shRNA-HuR glaucomatous animals treated with an MT intravitreal injection, whose beneficial effects on neurons have been described in the literature (Pedersen et al., 2009a,b; Pietrucha-Dutczak et al., 2017), were evaluated to better understand the role of HuR in glaucoma and the RGC stress response. We found that in the shRNA-control group, MT preserved the RGC count in both the central and peripheral retina; in contrast, MT was ineffective in shRNA-HuR animals, where consistent RGC death was detected in both retinal regions. Similarly, despite MT administration, the optic nerve displayed significant damage in the shRNA-HuR glaucomatous group at week 8 that was characterized by axon swelling and loss, myelin sheet disintegration, and marked inflammation, as evidenced by the infiltration of a large number of activated macrophages. The microglia infiltration is derivative of neurodegeneration itself and in glaucoma these cell may contribute to both propagation of neurodegeneration and modulation of its course (Wei et al., 2019). Of relevance, 8 weeks after AAV injection and 4 weeks after glaucoma induction and MT treatment, the RGC function measured by PhNR in shRNA-HuR rats was considerably impaired compared to both healthy and shRNA-control animals (−77 and −50%, respectively), and it was fully abolished at the end of the experiment in week 16 (−96 and −86%, respectively). Based on this finding, HuR is an endogenous neuroprotective factor in the retina; if HuR function is impaired, RGC function is irreversibly compromised at early stages after glaucoma onset, and it further declines, despite the presence of the neuroprotective MT. Our results are consistent with a previous study showing that *hur*^–/–^ knockout animals develop hypersensitivity of hippocampal neurons to oxidative and excitotoxic damage (Skliris et al., 2015).

According to our previous (Smedowski et al., 2018) and present evidence, we may infer that HuR loss within RGCs is a general feature of glaucomatous damage and that factors (i.e., genetic variability and drugs) blocking or decreasing HuR function/expression at the retinal level likely contribute to accelerating glaucoma induced RGC loss and dysfunction. Interestingly, MT exerts a moderate but significant neuroprotective effect on exogenous stress, at least in the short term after glaucoma induced damage, according to the role of MT as an acute phase stress factor (Suemori et al., 2006; Ling et al., 2016). In both, the central and peripheral retinal regions, the MT-treated shRNA-control group indeed displayed RGC counts similar to those in the healthy group, together with preservation of retinal function until 8 weeks after glaucoma induction. Moreover, histological optic nerve studies showed that MT treatment protected shRNA-control rats from glaucoma induced axon damage and loss.

According to previous observations (Wang et al., 2016; Ramirez et al., 2017; Adornetto et al., 2019; Wei et al., 2019; García-Bermúdez et al., 2021; Zhao et al., 2021), glaucomatous damage is accompanied by an increase in the number of Iba1-positive cells at the retinal level, an index of microglial infiltration and activation, which is stronger in the MT-treated shRNA-HuR group than in the MT-treated shRNA control group. In the latter group, the microglia seem to exhibit a diffuse localization, while in the former group, they are widespread but also accumulate in foci. By regulating metal ions and through its cysteine-rich structure, MT, whose physiological expression is increased by various stresses, acts as a neuroprotective and anti-inflammatory mediator through several pathways mainly linked to the redox stress response and apoptosis (Coyle et al., 2002; Inoue et al., 2009). More generally, the human eye is enriched in metallothioneins, which mediate antioxidant, anti-inflammatory, and neuroprotective endogenous mechanisms in various ocular tissues (Gonzalez-Iglesias et al., 2014). The finding that exogenous MT treatment is ineffective in shRNA-HuR-treated glaucomatous animals suggests that MT requires the presence of HuR or its downstream factors to properly exert its benefits. In this regard, several detoxifying enzymes, and anti-inflammatory factors whose expression is regulated by HuR are linked to MT-mediated defenses (Akbari, 2020). However, further studies will be needed to clarify these findings.

The current *in vitro* study supports our theory and confirms the importance of HuR in efficient endogenous and exogenous neuroprotection. In order to investigate the impact of *hur* silencing on B35 cell survival, we used two different settings of insults to analyze different conditions for cell death. Since HuR is known regulator of Hsp proteins system, we used heat preconditioning to specifically evaluate the impact of HuR silencing on Hsp proteins. NMDA insult was introduced, since excitotoxicity is one of the major mechanisms in neurodegeneration, also in the retina. Both Hsp proteins and excitotoxicity play role in RGC death and survival. As expected, rat neuronal-like B-35 cells exposed to either temperature preconditioning or heat shock upregulated HuR expression as a defense mechanism; a concomitant increase in Hsp70 protein expression was observed as a downstream consequence of HuR activation. Notably, *hur* silencing limits the cytoprotection provided by either MT pretreatment or temperature preconditioning in rat neuronal-like B-35 cells exposed to heat shock; the trend for Hsp70 expression is similar to that of the HuR content. Interestingly, as a preventive strategy, MT treatment is more effective than temperature preconditioning, and it also better preserves the cellular projections. However, MT-induced protection is abolished in shRNA-HuR-treated cells. No changes in the HuR content were observed, when MT was administered during recovery after the insult in either shRNA-control- or shRNA-HuR-treated cells; instead, both groups showed HuR-independent upregulation of Hsp70.

Another cytotoxic stimulus, 24 h of NMDA treatment, did not affect the HuR content in the cytoplasm of B-35 cells, although the HuR protein seemed to be activated by chemical stress. Indeed, the downstream protein Hsp70 is upregulated in NMDA-treated shRNA-control cells in a HuR-dependent manner, since the stress-induced increase in Hsp70 expression is abolished in shRNA-HuR cells. This finding is consistent with previous observations that HuR content may not be modified, although the protein is activated (Marchesi et al., 2018). On the other hand, the decrease in the HuR content within the cytoplasm was accompanied by a concomitant decrease in Hsp70 protein levels in the same *hur*-silenced cells under normal conditions in the absence of stress. Additionally, following NMDA exposure, shRNA-HuR-treated cells presented higher levels of both ROS and 4-HNE, and they were more prone to cell death than shRNA-control-treated cells.

There are several limitations of our study that should be highlighted. The lack of commercially available RGC cell line resulted in need of applying other cellular model for the *in vitro* screening experiment. Since neuroblastoma is well established and widely described neuronal-like cell line, we used it in our initial testing. Other limitations include the RGC density evaluation using fixed-sampling protocol. We used high number of counting fields with the respect to superior-inferior RGC distribution differences, to provide the best possible representation of the retina using this method.

## 5. Conclusion

Based on our *in vitro* and *in vivo* findings, we conclude that HuR is essential for the efficient endogenous and exogenous neuroprotection of RGCs and that the induced alteration in the HuR content accelerates both the age-related and glaucoma-induced decreases in the RGC number and function, further confirming the key role of HuR in maintaining cellular homeostasis and its involvement in the pathogenesis of glaucoma.

## Data availability statement

The original contributions presented in this study are included in the article/supplementary material, further inquiries can be directed to the corresponding author.

## Ethics statement

The animal study was reviewed and approved by the Local Ethical Committee for Animal Research, Medical University of Silesia, Katowice, Poland.

## Author contributions

AP: design of the work, the acquisition and analysis of data, writing manuscript, and substantial review. JM and PR: acquisition and analysis of data and writing manuscript. SA: acquisition and analysis of data regarding electron microscopy, writing manuscript, and substantial review. XL: acquisition and analysis of data, writing manuscript, preparation of figures, and substantial review. MP-D: acquisition of data. JL-K and MA: conception, design of the work, writing manuscript, and substantial review. AS: conception, design of the work, the acquisition, analysis, and interpretation of data, preparation of figures, writing manuscript, and substantial review. All authors read and approved the final manuscript.

## Footnotes

1
http://imagej.nih.gov/ij/

## References

[B1] Abdelmohsen K. Kuwano Y. Kim H. Gorospe M. (2008). Posttranscriptional gene regulation by RNA-binding proteins during oxidative stress: implications for cellular senescence. *Biol. Chem.* 389 243–255. 10.1515/BC.2008.022 18177264 PMC8481862

[B2] Adornetto A. Russo R. Parisi V. (2019). Neuroinflammation as a target for glaucoma therapy. *Neural Regen. Res.* 14 391–394. 10.4103/1673-5374.245465 30539803 PMC6334605

[B3] Akbari G. (2020). Role of Zinc supplementation on ischemia/reperfusion injury in various organs. *Biol. Trace Elem. Res.* 196 1–9. 10.1007/s12011-019-01892-3 31828721

[B4] Almasieh M. Wilson A. Morquette B. Cueva Vargas J. di Polo A. (2012). The molecular basis of retinal ganglion cell death in glaucoma. *Prog. Retin. Eye Res.* 31 152–181. 10.1016/j.preteyeres.2011.11.002 22155051

[B5] Amadio M. Scapagnini G. Laforenza U. Intrieri M. Romeo L. Govoni S. (2008). Post-transcriptional regulation of HSP70 expression following oxidative stress in SH-SY5Y Cells: the potential involvement of the RNA-Binding Protein HuR. *Curr. Pharm. Des.* 14 2651–2658. 10.2174/138161208786264052 18991684

[B6] Ambrosio F. Coricello A. Costa G. Lupia A. Micaelli M. Marchesi N. (2021). Identification of compounds targeting HuD. Another brick in the wall of neurodegenerative disease treatment. *J. Med. Chem.* 64 9989–10000. 10.1021/acs.jmedchem.1c00191 34219450

[B7] Bowles K. Silva M. Whitney K. Bertucci T. Berlind J. Lai J. (2021). ELAVL4, splicing, and glutamatergic dysfunction precede neuron loss in MAPT mutation cerebral organoids. *Cell* 184 4547–63.e17. 10.1016/j.cell.2021.07.003 34314701 PMC8635409

[B8] Calkins D. Pekny M. Cooper M. Benowitz L. Calkins D. Benowitz L. (2017). The challenge of regenerative therapies for the optic nerve in glaucoma. *Exp. Eye Res.* 157 28–33. 10.1016/j.exer.2017.01.007 28153739 PMC5937264

[B9] Chi H. Chang H. Sang T. (2018). Neuronal cell death mechanisms in major neurodegenerative diseases. *Int. J. Mol. Sci.* 19:3082. 10.3390/ijms19103082 30304824 PMC6213751

[B10] Chrysostomou V. Trounce I. Crowston J. (2010). Mechanisms of retinal ganglion cell injury in aging and glaucoma. *Ophthalmic Res.* 44 173–178. 10.1159/000316478 20829641

[B11] Chun B. Cestari D. (2017). Advances in experimental optic nerve regeneration. *Curr. Opin. Ophthalmol.* 28 558–563. 28795960 10.1097/ICU.0000000000000417

[B12] Coyle P. Philcox J. Carey L. Rofe A. (2002). Metallothionein: the multipurpose protein. *Cell Mol. Life Sci.* 59 627–647. 10.1007/s00018-002-8454-2 12022471 PMC11337511

[B13] de Conti L. Baralle M. Buratti E. (2017). Neurodegeneration and RNA-binding proteins. *Wiley Interdiscip. Rev. RNA* 8:e1394. 10.1002/wrna.1394 27659427

[B14] della Santina L. Ou Y. (2017). Who’s lost first? Susceptibility of retinal ganglion cell types in experimental glaucoma. *Exp. Eye Res.* 158 43–50. 10.1016/j.exer.2016.06.006 27319294 PMC5161723

[B15] Doozandeh A. Yazdani S. (2016). Neuroprotection in glaucoma. *J. Ophthalmic Vis. Res.* 11 209–220. 10.4103/2008-322X.183923 27413504 PMC4926571

[B16] Fan N. Tan J. Liu X. (2019). Is “normal tension glaucoma” glaucoma? *Med. Hypotheses.* 1:133. 10.1016/j.mehy.2019.109405 31563827

[B17] Frade J. Ovejero-Benito M. (2015). Neuronal cell cycle: the neuron itself and its circumstances. *Cell Cycle* 14 712–720. 10.1080/15384101.2015.1004937 25590687 PMC4418291

[B18] Fry L. Fahy E. Chrysostomou V. Hui F. Tang J. van Wijngaarden P. (2018). The coma in glaucoma: retinal ganglion cell dysfunction and recovery. *Prog. Retin. Eye Res.* 65 77–92. 10.1016/j.preteyeres.2018.04.001 29631042

[B19] García-Bermúdez M. Freude K. Mouhammad Z. van Wijngaarden P. Martin K. Kolko M. (2021). Glial cells in glaucoma: friends, foes, and potential therapeutic targets. *Front. Neurol.* 12:624983. 10.3389/fneur.2021.624983 33796062 PMC8007906

[B20] Gauthier A. Liu J. (2016). Neurodegeneration and neuroprotection in glaucoma. *Yale J. Biol. Med.* 89 73–79. 27505018 PMC4797839

[B21] Gonzalez-Iglesias H. Alvarez L. García M. Petrash C. Sanz-Medel A. Coca-Prados M. (2014). Metallothioneins (MTs) in the human eye: a perspective article on the zinc-MT redox cycle. *Metallomics* 6 201–208. 10.1039/c3mt00298e 24419560

[B22] Gray D. Woulfe J. (2013). Structural disorder and the loss of RNA homeostasis in aging and neurodegenerative disease. *Front. Genet.* 4:149. 10.3389/fgene.2013.00149 23967011 PMC3743304

[B23] Himori N. Yamamoto K. Maruyama K. Ryu M. Taguchi K. Yamamoto M. (2013). Critical role of Nrf2 in oxidative stress-induced retinal ganglion cell death. *J. Neurochem.* 127 669–680. 10.1111/jnc.12325 23721546

[B24] Hu Y. (2016). Axon injury induced endoplasmic reticulum stress and neurodegeneration. *Neural Regen. Res.* 11 1557–1559. 10.4103/1673-5374.193225 27904477 PMC5116825

[B25] Inoue K. Takano H. Shimada A. Satoh M. (2009). Metallothionein as an anti-inflammatory mediator. *Mediat. Inflamm.* 2009:101659. 10.1155/2009/101659 19436762 PMC2679981

[B26] Kim C. Kang D. Lee E. Lee J. (2017). Long noncoding RNAs and RNA-binding proteins in oxidative stress, cellular senescence, and age-related diseases. *Oxid. Med. Cell Longev.* 2017:2062384. 10.1155/2017/2062384 28811863 PMC5547732

[B27] Laha B. Stafford B. Huberman A. (2017). Regenerating optic pathways from the eye to the brain. *Science* 356 1031–1034. 10.1126/science.aal5060 28596336 PMC6333302

[B28] Ling X. B. Wei H. Wang J. Kong Y. Wu Y. Guo J. (2016). Mammalian metallothionein-2A and oxidative stress. *Int. J. Mol. Sci.* 17:1483. 10.3390/ijms17091483 27608012 PMC5037761

[B29] Liu D. Greene L. (2001). Neuronal apoptosis at the G1/S cell cycle checkpoint. *Cell Tissue Res.* 305 217–228. 10.1007/s004410100396 11545259

[B30] Marchesi N. Thongon N. Pascale A. Provenzani A. Koskela A. Korhonen E. (2018). Autophagy stimulus promotes early HuR protein activation and p62/SQSTM1 protein synthesis in ARPE-19 cells by triggering Erk1/2, p38MAPK, and JNK kinase pathways. *Oxid. Med. Cell Longev.* 2018:4956080. 10.1155/2018/4956080 29576851 PMC5822911

[B31] Masuda K. Kuwano Y. Nishida K. Rokutan K. (2012). General RBP expression in human tissues as a function of age. *Ageing Res Rev.* 11 423–431. 10.1016/j.arr.2012.01.005 22326651

[B32] Milani P. Amadio M. Laforenza U. Dell’Orco M. Diamanti L. Sardone V. (2013). Posttranscriptional regulation of SOD1 gene expression under oxidative stress: potential role of ELAV proteins in sporadic ALS. *Neurobiol. Dis.* 60 51–60. 10.1016/j.nbd.2013.08.005 23969235

[B33] Moore D. Goldberg J. (2010). Four steps to optic nerve regeneration. *J. Neuro Ophthalmol.* 30 347–360. 10.1097/WNO.0b013e3181e755af 21107123

[B34] Naguib S. Backstrom J. Gil M. Calkins D. Rex T. (2021). Retinal oxidative stress activates the NRF2/ARE pathway: an early endogenous protective response to ocular hypertension. *Redox. Biol.* 42:101883. 10.1016/j.redox.2021.101883 33579667 PMC8113046

[B35] Nakazawa T. Fukuchi T. (2020). What is glaucomatous optic neuropathy? *Jpn. J. Ophthalmol.* 64 243–249. 10.1007/s10384-020-00736-1 32394134

[B36] Nuschke A. Farrell S. Levesque J. Chauhan B. (2015). Assessment of retinal ganglion cell damage in glaucomatous optic neuropathy: axon transport, injury and soma loss. *Exp. Eye Res.* 141 111–124. 10.1016/j.exer.2015.06.006 26070986

[B37] Osborne N. (2008). Pathogenesis of ganglion “cell death” in glaucoma and neuroprotection: focus on ganglion cell axonal mitochondria. *Prog. Brain Res.* 173 339–352. 10.1016/S0079-6123(08)01124-2 18929120

[B38] Osborne N. del Olmo-Aguado S. (2013). Maintenance of retinal ganglion cell mitochondrial functions as a neuroprotective strategy in glaucoma. *Curr. Opin. Pharmacol.* 13 16–22. 10.1016/j.coph.2012.09.002 22999653

[B39] Ouyang X. Yang J. Hong Z. Wu Y. Xie Y. Wang G. (2020). Mechanisms of blue light-induced eye hazard and protective measures: a review. *Biomed. Pharmacother.* 130:110577. 10.1016/j.biopha.2020.110577 32763817

[B40] Pascale A. Govoni S. (2012). The complex world of post-transcriptional mechanisms: Is their deregulation a common link for diseases? Focus on ELAV-like RNA-binding proteins. *Cell. Mol. Life Sci.* 69 501–517. 10.1007/s00018-011-0810-7 21909784 PMC11114966

[B41] Pedersen M. Jensen R. Pedersen D. Skjolding A. Hempel C. Maretty L. (2009a). Metallot. hionein-I+II in neuroprotection. *BioFactors* 35 315–325. 10.1002/biof.44 19655389

[B42] Pedersen M. Larsen A. Stoltenberg M. Penkowa M. (2009b). Cell death in the injured brain: roles of metallothioneins. *Prog. Histochem. Cytochem.* 44 1–27. 10.1016/j.proghi.2008.10.002 19348909

[B43] Penkowa M. (2006). Metallothioneins are multipurpose neuroprotectants during brain pathology. *FEBS J.* 273 1857–1870. 10.1111/j.1742-4658.2006.05207.x 16640552

[B44] Pérez de Lara M. Santano C. Guzmán-Aránguez A. Valiente-Soriano F. Avilés-Trigueros M. Vidal-Sanz M. (2014). Assessment of inner retina dysfunction and progressive ganglion cell loss in a mouse model of glaucoma. *Exp. Eye Res.* 122 40–49. 10.1016/j.exer.2014.02.022 24631335

[B45] Pietrucha-Dutczak M. Amadio M. Govoni S. Lewin-Kowalik J. Smedowski A. (2018). The role of endogenous neuroprotective mechanisms in the prevention of retinal ganglion cells degeneration. *Front. Neurosci.* 12:834. 10.3389/fnins.2018.00834 30524222 PMC6262299

[B46] Pietrucha-Dutczak M. Smedowski A. Liu X. Matuszek I. Varjosalo M. Lewin-Kowalik J. (2017). Candidate proteins from predegenerated nerve exert time-specific protection of retinal ganglion cells in glaucoma. *Sci. Rep.* 7:14540. 10.1038/s41598-017-14860-5 29109409 PMC5673995

[B47] Quan L. Uyeda A. Muramatsu R. (2022). Central nervous system regeneration: the roles of glial cells in the potential molecular mechanism underlying remyelination. *Inflamm. Regen.* 42 1–12. 10.1186/s41232-022-00193-y 35232486 PMC8888026

[B48] Ramirez A. de Hoz R. Salobrar-Garcia E. Salazar J. Rojas B. Ajoy D. (2017). The Role of microglia in retinal neurodegeneration: Alzheimer’s disease, parkinson, and glaucoma. *Front. Aging Neurosci.* 9:214. 10.3389/fnagi.2017.00214 28729832 PMC5498525

[B49] Sanna M. Ghelardini C. Galeotti N. (2017). HuD-mediated distinct BDNF regulatory pathways promote regeneration after nerve injury. *Brain Res.* 1659 55–63. 10.1016/j.brainres.2017.01.019 28111162

[B50] Sanna M. Peroni D. Mello T. Ghelardini C. Quattrone A. Galeotti N. (2016). Increase of neurofilament-H protein in sensory neurons in antiretroviral neuropathy: evidence for a neuroprotective response mediated by the RNA-binding protein HuD. *Pharmacol. Res.* 111 23–33. 10.1016/j.phrs.2016.05.026 27238228

[B51] Sanna M. Quattrone A. Mello T. Ghelardini C. Galeotti N. (2014). The RNA-binding protein HuD promotes spinal GAP43 overexpression in antiretroviral-induced neuropathy. *Exp. Neurol.* 261 343–353. 10.1016/j.expneurol.2014.05.017 24861443

[B52] Sanz-Morello B. Ahmadi H. Vohra R. Saruhanian S. Freude K. Hamann S. (2021). Oxidative stress in optic neuropathies. *Antioxidants* 10:1538. 10.3390/antiox10101538 34679672 PMC8532958

[B53] Skliris A. Papadaki O. Kafasla P. Karakasiliotis I. Hazapis O. Reczko M. (2015). Neuroprotection requires the functions of the RNA-binding protein HuR. *Cell Death Differ.* 22 703–718. 10.1038/cdd.2014.158 25301069 PMC4392069

[B54] Smedowski A. Liu X. Podracka L. Akhtar S. Trzeciecka A. Pietrucha-Dutczak M. (2018). Increased intraocular pressure alters the cellular distribution of HuR protein in retinal ganglion cells – A possible sign of endogenous neuroprotection failure. *Biochim. Biophys. Acta Mol. Basis Dis.* 1864 296–306. 10.1016/j.bbadis.2017.10.030 29107807

[B55] Solley W. Sternberg P. (1999). Retinal phototoxicity. *Int. Ophthalmol. Clin.* 39 1–12. 10.1097/00004397-199903920-00002 10343921

[B56] Suemori S. Shimazawa M. Kawase K. Satoh M. Nagase H. Yamamoto T. (2006). Metallothionein, an endogenous antioxidant, protects against retinal neuron damage in mice. *Invest. Ophthalmol. Vis. Sci.* 47 3975–3982. 10.1167/iovs.06-0275 16936113

[B57] Szymanski J. Wang H. Jamison J. DeGracia D. (2013). HuR function and translational state analysis following global brain ischemia and reperfusion. *Transl. Stroke Res.* 4 589–603. 10.1007/s12975-013-0273-2 24323414 PMC3864748

[B58] Upadhyay M. Milliner C. Bell B. Bonilha V. (2020). Oxidative stress in the retina and retinal pigment epithelium (RPE): role of aging, and DJ-1. *Redox Biol.* 37:101623. 10.1016/j.redox.2020.101623 32826201 PMC7767746

[B59] Varadarajan S. Huberman A. (2018). Assembly and repair of eye-to-brain connections. *Curr. Opin. Neurobiol.* 53 198–209. 10.1016/j.conb.2018.10.001 30339988 PMC6504177

[B60] Vernazza S. Oddone F. Tirendi S. Bassi A. Petrus-Reurer S. Ortín-Martínez A. (2021). Molecular sciences risk factors for retinal ganglion cell distress in glaucoma and neuroprotective potential intervention. *Int. J. Mol. Sci.* 22:7994. 10.3390/ijms22157994 34360760 PMC8346985

[B61] Völgyi B. (2020). Molecular biology of retinal ganglion cells. *Cells* 9 596–601. 10.3390/cells9112483 33203148 PMC7697858

[B62] Wang H. Anggraini F. Chen X. Degracia D. (2017). Embryonic lethal abnormal vision proteins and adenine and uridine-rich element mRNAs after global cerebral ischemia and reperfusion in the rat. *J. Cereb. Blood Flow Metab.* 37 1494–1507. 10.1177/0271678X16657572 27381823 PMC5453468

[B63] Wang J. Chen S. D. Zhang X. Jonas J. (2016). Retinal microglia in glaucoma. *J. Glaucoma* 25 459–465. 10.1097/IJG.0000000000000200 25646715

[B64] Wang W. Yang X. Cristofalo V. Holbrook N. Gorospe M. (2001). Loss of HuR is linked to reduced expression of proliferative genes during replicative senescence. *Mol. Cell Biol.* 21 5889–5898. 10.1128/MCB.21.17.5889-5898.2001 11486028 PMC87308

[B65] Wei X. Cho K. Thee E. Jager M. Chen D. (2019). Neuroinflammation and microglia in glaucoma: time for a paradigm shift. *J. Neurosci. Res.* 97 70–76. 10.1002/jnr.24256 29775216 PMC6239948

[B66] You Y. Gupta V. Li J. Klistorner A. Graham S. (2013). Optic neuropathies: characteristic features and mechanisms of retinal ganglion cell loss. *Rev. Neurosci.* 24 301–321. 10.1515/revneuro-2013-0003 23612594

[B67] Youssef P. Sheibani N. Albert D. (2011). Retinal light toxicity. *Eye* 25 1–14. 10.1038/eye.2010.149 21178995 PMC3144654

[B68] Zhang L. Feng H. Jin Y. Zhan Y. Han Q. Zhao X. (2021). Long non-coding RNA LINC01119 promotes neuropathic pain by stabilizing BDNF transcript. *Front. Mol. Neurosci.* 14:673669. 10.3389/fnmol.2021.673669 34234645 PMC8255623

[B69] Zhao X. Sun R. Luo X. Wang F. Sun X. (2021). The interaction between microglia and macroglia in glaucoma. *Front. Neurosci.* 15:610788. 10.3389/fnins.2021.610788 34121982 PMC8193936

